# The Birth of a Great Profession

**Published:** 1921-03-12

**Authors:** 


### SURGICAL EDUCATION IN THE SIXTEENTH CENTURY.

### The Birth of a Great Profession.

In the second Vicary lecture, which Sir D'Arcy Power delivered at the Royal College of Surgeons, and published later in the *British Journal of Surgery*, he laid before his audience facts of unusual interest. In modern times one is so apt, in a scientific profession, to think that the greater part of our present-day practice is, in the truest sense, new. Yet if we scan the results of the sixteenth century there is no doubt that our fathers were indeed alive to their surroundings.

Vicary, after whom these historical lectures are named, was foremost among that band of pioneer surgeons who, in the reign of Henry VIII., desired to see their calling advanced from a trade to a profession. Although not immediately successful, it is largely owing to the efforts of these men that the surgeon owes his present social position in this country.

### The Turning Point.

Following the Wars of the Roses, and up to 1485, England had been in an almost chaotic state, until the later accession of King Henry VII. and his marriage with Lady Elizabeth of York brought peace to the land, and with it a phase of general advance and reconstruction, by which surgery, together with most of the arts, was duly affected. London had always supported a small body of surgeons independently of the barbers. These men performed the same relative duties as the consulting surgeons of to-day, although the method of their practice was slightly different. In the customary arrangement they stayed with their patient until he was cured, and were in this capacity generally attached to the higher nobility. During active warfare the surgeons in this way made a good, if precarious living, but the advent of peace under Henry VII. caused them generally to reconsider their position. Much of the old nobility had been destroyed, and the newly formed middle-classes had now to be catered for. With this object, in 1493, they entered into an agreement with the Barbers' Company, and therein is contained a special clause dealing with the examination of surgeons. The licence then granted to Robert Anson is still in existence, and it is seen that even at this early period a special examiner was appointed to conduct a public examination in surgery.

Nevertheless, this fraternity of surgeons dealt at that time with the practising surgeon rather than the apprentice or student. The latter was still under the care of the Barbers' Company, for the London guilds, even before they became chartered companies, had always taken an interest in the education of their members, and had endeavoured by a system of apprenticeship to provide satisfactory craftsmen. But the Company of Barbers was formally united with the surgical fraternity by Act of Parliament in 1540, and Vicary was elected Master of the United Company at the end of John Pen's term of office. He acted in this capacity from 1540 to 1542, and his position at the head of the Union has fortunately been commemorated for us in the well-known picture by Holbein.

After the Union had once been established the Company set about solving a variety of problems. These were, in character, curiously like those which face the medical world of to-day. There was the internal problem concerning the better education of the professions and the external, implying the better education of the public to appreciate the proper treatment of disease. For the first a fairly adequate system of education was being evolved, but so long as the surgeon remained subordinate to the physician a great handicap was experienced. This subordination remained for many years—and, as is only too true, the education of the public is perhaps even yet incomplete.

### Apprenticeship.

It is surprising to learn that these early apprentices were frequently, in fact generally, drawn from the lower classes, the father's occupation being frequently given as "labourer." There were exceptional instances, such as the Arris family, in whom the profession was served for three generations. But, as Thomas Gale says in "An Institution of a Chirurgion," "few that have wel brought up their sonne will put him to the arte, because it is accounted so beggerly and vile." As late as 1570 Sir Humphrey Gilbert protests that "we have verie few good Chirurgions yf any at all. By reason that cirurgerie is not now learned in any other place than in a Barbors shoppe."

The average number of apprentices presented annually at the Barbers' Hall was 150, so that, indentures lasting seven years, there were always about 1,000 apprentices belonging to the Barber Surgeons alone, and other City companies had them in proportion. Fortunately—for these young men needed a tight hand over them—the Barber Surgeons had ample disciplinary powers, for they could whip or imprison them at will.

### Surgical Education.

But generally speaking the system of education appears to have been by simple lecturing. After the formation of the United Company each member of the Guild was pledged under a fine of twenty shillings either to lecture himself or to provide a substitute. There are no written records left, but we know that these men exercised a great and needed influence upon the surgical ethics of the day. Probably their lectures dealt chiefly with their own experience, but, with none of their writings discoverable, even Morested, Bradwardyn, Ferris, Keble, and other great surgeons of the time are now little more than names to us.

Even the study of anatomy as then followed was more remarkable for the devious methods by which a supply of human material was obtained than for any great eccentricity in teaching. Mostly the dissections were carried out upon the bodies of those "condempned, adjudged and put to deathe for feloni." But the most potent influence not only upon all anatomical study, but upon the Company itself, arose with the first appointment of a Reader of Anatomy. Sir D'Arcy Power traces the early appointments as follows:

"A vacancy for a Reader of Anatomy occurred about 1546, and the Company adopted the wise policy of choosing the best possible man for the post. They elected, no doubt by the advice of Vicary and on the recommendation of Dr. Butts, Dr. John Caius, a Cambridge graduate, aged thirty-six. Caius had lately returned from Italy, where he had been a fellow-lodger with Vesalius at Padua and an acquaintance of Realdus Columbus, the two great anatomists of their age. He was also a competent Greek scholar, and had made a special study of Galen, hunting through the great libraries of Italy for accurate texts. In 1544 he issued his edition of the "De Medendi Methodo" and other works of Galen which had not previously been published. No better choice could have been made by a young and virile society which included a large uncultured element amongst its members. Caius was a grave and learned man, a stickler for etiquette,

**Surgical Education in the 16th Century—(*continued*).**

and a bachelor. He lived in the immediate neighbourhood of the Barber Surgeons' Hall in Monkwell Street, for he had a house in St. Bartholomew's Hospital, and could thus devote sufficient time and energy to his work. He held office until 1563, and was succeeded by Dr. Cunningham. His long tenure of the lectureship left a permanent mark upon the teaching. The Reader of the Anatomy was ever afterwards a young University graduate. The post was well paid, and many of its holders subsequently achieved fame."

This is but one of many similar instances, but well illustrates a great step forward not only in learning, but also in characterisation of a surgeon's standing.

The Education of the Public.

The lecturer gives us also a typical picture of the second problem before the United Company. The public attitude towards skilled medicine was indeed complex, and is best judged from the following outline :

The education of the public to appreciate the value of better surgical treatment proved to be a more difficult task than the education of the surgeon himself. Surgery had reached a very low ebb at the beginning of the sixteenth century, for in 1511 an Act of Parliament for the appointing of physicians and surgeons recites that "the science and cunning of Physic and Surgery is daily within this Realm exercised by a great multitude of ignorant persons of whom the greater part have no insight in the same nor in any other kind of learning. Some also can no letters from the Book, so far forth that common Artificers as Smiths, Weavers and Women boldly and accustomably take upon them great Cures . . . to the great infamy of the Faculty and the greivous Hurt, damage and destruction of the King's Liege people most especially of them that cannot discern the uncunning from the cunning." This is confirmed by Clowes some years later, who says "that many in these days do take upon them to intermeddle and practise in this art, wherein they were never trained nor had any experience; of the which a great number be shameless in countenance, lewd in disposition and brutish in judgement, which do forsake their honest trade whereunto God hath called them and do daily rush into physic and surgery." The state of surgery, indeed, was nearly such as to warrant the advice given to a surgeon by William of Salicet in 1275, that "a wise surgeon will do well to refrain from stealing anything while he is actually in attendance and will not employ notoriously bad characters as his assistants because such actions are apt to lessen the confidence of the patient and may thus spoil an otherwise good operation."

In face of this it is not surprising that much pioneer work remained. But fortunately the same influences were already improving the knowledge and education of the surgeon himself, gradually achieved a public enlightenment also. The changes thus produced were impossibly slow, but they materialised nevertheless, and in many ways the period considered stands as the starting-point in the health education of England.

